# Establishment of new energy supply construction inspired by the defensive mechanism of *Pheropsophus occipitalis*

**DOI:** 10.3389/fchem.2026.1831087

**Published:** 2026-10-20

**Authors:** Lin Wang, Yun Zhang, Fanghan Che, Xinyi Yao, Jiajun Yu, Weiping Li, Ruoshui Wang, Ruochun Yin

**Affiliations:** 1 School of Life Science, Anhui University, Hefei, Anhui, China; 2 State Key Laboratory of Microbial Diversity and Innovative Utilization, Institute of Microbiology, Chinese Academy of Sciences, Beijing, China; 3 College of Life Sciences, University of Chinese Academy of Sciences, Beijing, China; 4 School of Biotechnology, Jiangnan University, Wuxi, China

**Keywords:** hydrogen peroxide, hydroquinone, laccase, new energy-supply construction, *Pheropsophus occipitalis*

## Abstract

The defensive chemical system of *Pheropsophus occipitalis* has long been a research focus on biomimetic catalysis and energy applications, yet the biosynthetic origin of hydrogen peroxide (H_2_O_2_) in its defensive glands remains unresolved. This work aimed to decipher the underlying catalytic mechanism and develop a novel biomimetic energy supply strategy. Electron paramagnetic resonance (EPR) spectroscopy and molecular docking were employed to verify the radical-mediated pathway of laccase-catalyzed hydroquinone oxidation. Laccase and catalase were co-immobilized on cerium oxide–titanium dioxide composite supports to enhance catalytic stability and efficiency. A biomimetic microchannel catalyst bed coupled with a pulsed cascade booster system was further constructed, inspired by the compartmentalized anatomy of *P. occipitalis*. We demonstrate for the first time that laccase catalyzes hydroquinone oxidation to generate H_2_O_2_ via radical intermediates, and reveal a triple synergistic catalytic cycle consisting of oxygen regeneration, hydrogen element turnover, and cerium redox shuttle. The immobilized laccase yields a relative H_2_O_2_ concentration of 191.79%, corresponding to a 9.2-fold improvement over free laccase. The integrated biomimetic system realizes on-demand in situ generation and utilization of H_2_O_2_. This study advances the mechanistic understanding of the *P. occipitalis* chemical defense system, and provides a novel, low-noise and eco-friendly technical route for the development of portable energy supply systems.

## 1 Introduction

Currently, three primary energy-supply systems dominate the global landscape: lithium- ion batteries, fuel cells, and internal combustion engines (Table 1). Nevertheless, as the most widely used power source, lithium-ion batteries have a relatively low energy density, which imposes significant limitations on their long-term operation. Although fuel cell technology has advanced considerably and achieved high energy and power densities, high manufacturing costs and harmful substrate emissions remain critical unresolved challenges. Internal combustion engines possess the highest energy and power densities, making them theoretically optimal power systems. However, their inherent drawbacks, including noise pollution, low energy efficiency, and harmful exhaust emissions, remain intractable in the short term. Consequently, the development of novel power systems that combine high power density, high energy density, and environmental friendliness has emerged as a key bottleneck that hinders practical applications.

**TABLE 1:** Comparison of energy and power densities of different power systems (Li et al., 2004).

Power system	Energy density (MJ/kg)	Power density (kW/kg)
Internal combustion engine	0.8∼10	0.09∼0.9
Lithium-ion battery	0.03∼0.65	0.008∼0.3
Fuel cell	0.4∼7	0.01∼0.18
H_2_O_2_ (95%)	1.2∼1.6	0.006∼0.15

Against the background of global energy decarbonization, hydrogen is widely recognized as a core zero-carbon energy carrier with high energy density and clean end-use emissions, showing great application potential in transportation, portable power supply, and aerospace propulsion (Ali et al., 2022c; Basheer and Ali, 2019). Currently, global hydrogen production is still dominated by fossil fuel reforming, whereas green hydrogen production through water splitting driven by renewable energy has gained extensive attention. Nanocatalysts represented by green-synthesized nanoparticles and metal oxide radiation-catalytic systems have effectively improved water decomposition efficiency; however, the storage, transportation, and safety risks of gaseous hydrogen remain key bottlenecks limiting its large-scale application in portable and distributed power scenarios (Ali et al., 2022a; Ali et al., 2022c; Basheer and Ali, 2019; Ragauskas et al., 2020).

Advanced functional catalytic materials, such as nanostructured metal oxides and metallic alloys, have emerged as a prominent research focus in radiation-driven hydrogen production because of their tailorable surface chemistry, abundant defect sites, and highly efficient interfacial energy transfer. Ali et al. (2021) have demonstrated that nanostructured zirconia (ZrO_2_) significantly boosts the efficiency of water γ-radiolysis. Specifically, the hydrogen yield in the liquid-phase system is 6.0–7.0-fold higher than that in the adsorbed-phase system, attaining 19.6 molecules per 100 eV for 50 nm–70 nm particles at 673 K (Ali et al., 2021). Building on this foundation, incorporating n-hexane to form a ternary water–n-hexane–nano-ZrO_2_ system elevates the hydrogen yield to 188.5 molecules per 100 eV (Ali et al., 2023a). For nanostructured alumina (Al_2_O_3_), a pronounced size dependence of radiation-catalytic activity was identified: 5 nm nanoparticles exhibit 1.4–1.6-fold higher hydrogen yield relative to 40 nm–50 nm counterparts, with a maximum yield of 5.97 molecules per 100 eV (Ali et al., 2023b). Among metallic alloy catalysts, the Zr1%Nb alloy achieves a hydrogen yield of up to 437.4 molecules per 100 eV when applied to seawater radiolysis (Ali et al., 2022b). Despite continuous improvements in hydrogen production efficiency enabled by these advanced materials, the inherent bottlenecks of gaseous hydrogen regarding its storage, transportation, and safety remain largely unaddressed at the fundamental level, constraining its large-scale deployment in portable and distributed power supply scenarios.

As a liquid hydrogen-based energy alternative, hydrogen peroxide (H_2_O_2_) exhibits storage safety and system simplicity for portable and distributed power supplies. As a monopropellant, hydrogen peroxide undergoes decomposition without requiring external oxygen, enabling the design of simple and reliable propulsion systems. Experimental studies have demonstrated that power systems using 70% hydrogen peroxide exhibit a power density 4–5 times higher than that of lithium-ion battery-motor systems. Additionally, increasing the concentration to 100% further enhances the power density by an order of magnitude, highlighting its enormous development potential (Gogola et al., 2002). The conventional industrial production method for hydrogen peroxide, the anthraquinone process, has the disadvantages of high energy consumption and severe waste generation. The catalytic reduction of water to produce hydrogen peroxide has garnered increasing attention as a promising green alternative. However, though water is a cheaper and more readily available hydrogen source compared to organic reagents, it is inherently a poor hydrogen donor due to the high O–H bond dissociation energy of water molecules (homolytic bond dissociation energy: 492 kJ·mol^-1^). Consequently, achieving efficient H_2_O_2_ photosynthesis using only molecular oxygen and water is of great significance; however, it remains a formidable challenge.

*Pheropsophus occipitalis*, a coleopteran species, has garnered global attention owing to its defense mechanisms and anatomical features. This species can explosively spray irritating gases and quinone compounds at near-boiling temperatures (∼100 °C) when threatened by predators (Aneshansley et al., 1969). Since its discovery in the 1960s, the defense mechanisms of *P. occipitalis* have been extensively investigated. Pioneering work by Eisner et al. elucidated the anatomical basis and physiological mechanisms underlying this defense mechanism through comprehensive anatomical and experimental investigations (Aneshansley et al., 1969; Dean et al., 1990; Eisner and Aneshansley, 1982). Their research revealed that specialized glands produce and store hydrogen peroxide and hydroquinone in separate reservoirs. Upon predator encounter, muscular contractions facilitate the rapid mixing of these precursors in a dedicated reaction chamber, where enzymatic catalysis generates both noxious quinone compounds and propellant gases. Recent studies have provided additional insights into biochemical pathways, demonstrating that m-cresol and oxygen undergo NADPH-catalyzed conversion to dimethyl-p-phenol and H_2_O, although the precise origin of hydrogen peroxide remains to be fully elucidated (Attygalle et al., 2020). Furthermore, Lehmann identified the presence of laccase in *P. occipitalis* and established its crucial role in benzoquinone biosynthesis, adding another layer of complexity to our understanding of this remarkable chemical defense system (Lehmann, 2015).

Multicopper oxidases, including laccase and tyrosinase, were also identified in *P. occipitalis* (Lehmann, 2015). Laccase (benzenediol: oxygen oxidoreductase, EC1.10.3.2), a prominent member of this family, features an active center composed of four Cu^2+^ ions. The T1 site, which contains a single Cu^2+^ ion, primarily facilitates dehydrogenation and de-electronation. Upon accepting an electron from substrates, the Cu^2+^ at the T1 site is reduced to Cu^1+^, which subsequently transfers the electron to downstream acceptors and reverts to its thermally stable Cu^2+^ state. The T2/T3 sites, which comprise a tri-nuclear copper cluster, are primarily responsible for the reduction of molecular oxygen. These sites receive four electrons from the T1 site and catalyze the four-electron reduction of O_2_ to H_2_O (Solomon et al., 1996; ten Have and Teunissen, 2001; Lee et al., 2002; Solomon et al., 2014). This electron transfer mechanism supports the catalytic versatility of laccases and related multicopper oxidases in biological systems.

Cerium is a rare-earth element belonging to the lanthanide series. It exists in the form of free metal or oxides and can form a reversible redox couple between cerium(III) oxide (Ce_2_O_3_) and cerium(IV) oxide (CeO_2_) (Hu et al., 2006). As a typical rare earth mesoporous material, nano-ceria possesses chemical and physical properties similar to those of cerium oxide but exhibits a larger specific surface area and abundant oxygen vacancies (Thrall and Pounds, 2007). It also exhibits superoxide dismutase-mimetic activity and radical regulation capability, which can effectively promote O_3_ radical conversion and hydrogen peroxide generation (Heckert et al., 2008). Titanium dioxide, a common photosensitive semiconductor material, assists the activation of oxygen molecules and induces radical formation under mild conditions (Veziroglu et al., 2024). When combined with cerium oxide, they form stable and efficient composite catalytic interfaces. Both materials exhibit excellent biocompatibility and structural tunability, making them ideal carriers for enzyme immobilization and biomimetic catalytic systems. They provide crucial material support for mimicking the cascade catalytic reactions in *P*. *occipitalis* and for constructing microchannel network catalyst beds.

Building on the documented hydroxyl radical generation from catechol and its derivatives via laccase catalysis (Chen et al., 2017), along with laccase-mediated H_2_O oxidation to hydrogen peroxide (Pita et al., 2014), we hypothesize that H_2_O_2_ stored in *P. occipitalis* defensive glands is derived from laccase-catalyzed oxidation of hydroquinone and its derivatives. However, three critical research gaps remain unaddressed: a) Mechanistic gap: the exact biosynthetic pathway of H_2_O_2_ in *P. occipitalis* lacks experimental validation, and the radical-mediated reaction process and underlying driving mechanism remain unclear. b) Technical gap: free laccase has poor stability and low H_2_O_2_ production efficiency, and existing enzyme immobilization strategies rarely integrate the auxiliary catalytic function of carrier materials for synergistic reaction enhancement. c) Application gap: conventional H_2_O_2_-based power systems rely on high-pressure storage of concentrated H_2_O_2_, with prominent safety risks, and biomimetic catalytic systems tailored for portable energy scenarios remain insufficient.

To bridge these gaps, this study first verifies via electron paramagnetic resonance (EPR) detection and molecular docking that laccase catalyzes hydroquinone oxidation to generate H_2_O_2_ via radical intermediates, clarifying the long-unresolved origin of H_2_O_2_ in the *P. occipitalis* defense system. On this basis, laccase and catalase are co-immobilized on cerium oxide–titanium dioxide composite supports to improve enzyme stability and catalytic efficiency, with the Ce^4+^/Ce^3+^ redox cycle acting as an electron shuttle for auxiliary catalysis. Finally, inspired by the compartmentalized anatomy of *P. occipitalis*, a biomimetic fractal microchannel catalyst bed and pulsed cascade booster system are constructed to achieve *in situ* H_2_O_2_ generation and utilization. This study provides new insights into insect chemical defense mechanisms and a novel technical route for developing low-noise, high-efficiency, and clean portable energy systems (Li et al., 2004).

## 2 Materials and methods

### 2.1 Materials

Laccase was obtained from Sigma-Aldrich (United States of America). Catalase, H_2_O_2_ detection kit (based on titanium sulfate colorimetry), cerium oxide (CeO_2_), hydroquinone, and cyanuric acid chloride were procured from Hefei BomeiBiotechnology Co., Ltd. (Anhui, China). 2,4-Bis(O-methoxypolyethylene glycol)-6-chloro-s-triazine (mPEG, CP) was sourced from Sinopharm Chemical Reagent Co., Ltd. The spin-trapping reagent 5,5-dimethyl-1-pyrroline N-oxide (DMPO, >99.0% purity) was purchased from Dojindo International. All chemicals were of analytical grade and used as received, unless otherwise specified.

### 2.2 The biologic mechanism of hydrogen peroxide biosynthesis

#### 2.2.1 Catalytic reaction and hydrogen peroxide detection

A 1% hydroquinone solution was mixed with different types of laccases (free, modified, and immobilized) and stirred continuously at 4 °C for 24 h. The concentration of hydrogen peroxide was quantitatively determined using a hydrogen peroxide detection kit according to the manufacturer’s protocol (titanium sulfate colorimetric method).

#### 2.2.2 Capture of radicals

A 10% hydroquinone solution was catalyzed by free laccase. The reaction proceeded at room temperature for either 5 or 15 min, after which the sample was rapidly quenched in liquid nitrogen to preserve the transient intermediates for subsequent EPR analysis. The freeze-trapped EPR spectra were acquired using a Bruker X-band EMX EPR spectrometer operating at 20 K.

For the spin-trapping experiments, a reaction mixture containing 10% hydroquinone solution and free laccase was maintained at room temperature for 3 min–4 min. Subsequently, the spin-trapping reagent DMPO was rapidly introduced to capture the radical species. EPR measurements were performed at room temperature to characterize the trapped radicals.

#### 2.2.3 Molecular docking

Water molecules and extraneous ligands were eliminated from the protein structure using PyMOL software. The preprocessed compounds were designated as ligands, and the protein served as the receptor. Molecular docking simulations were performed using AutoDockTools 1.5.7 with default parameters, from which the two structures exhibiting the lowest binding energies were selected for further analysis. Molecular interactions were subsequently analyzed and visualized using PyMOL-2.1 in conjunction with AutoDockTools 1.5.7.

### 2.3 Laccase and catalase modification and immobilization

#### 2.3.1 Activation of mPEG and modification of enzymes

Because mPEG lacks direct reactivity with enzyme functional groups, cyanuric acid chloride was utilized as an activating agent for mPEG functionalization. Additionally, 5.5 g of cyanuric acid chloride, 50 g of mPEG, 5 g of molecular sieve 5A, and 8.7 g of anhydrous sodium carbonate in anhydrous benzene were stirred for 12 h at room temperature. The reaction mixture was centrifuged, and the supernatant was collected for further purification. Purification via cyclic precipitation with anhydrous ether and dissolution with anhydrous benzene until washing led to no characteristic absorption at 241 nm. The purified product was lyophilized to yield activated mPEG, which was stored at 4 °C. Enzyme modification with activated mPEG was performed according to a previously established protocol (Daba et al., 2013).

#### 2.3.2 Characterization of transmission electron microscopy (TEM)

To prepare the enzyme–cerium oxide complexes, 100 mg of each modified enzyme (laccase and catalase) was individually mixed with 100 mg of cerium oxide nanoparticles in 1 mL of distilled water. The mixtures were continuously stirred for 12 h at room temperature, followed by centrifugation at 10,000 × *g* for 10 min to collect the precipitates. The obtained samples were homogeneously dispersed in absolute ethanol via ultrasonication for 15 min. Ten microliters of each suspension were deposited onto a 300-mesh copper grid and air-dried at room temperature. The samples were then analyzed using a FEI Tecnai G2 F30 TEM operated at 200 kV.

#### 2.3.3 Characterization of X-ray photoelectron spectroscopy (XPS)

A homogeneous mixture consisting of 100 mg of modified laccase, 100 mg of catalase, and 100 mg of cerium oxide in 1 mL distilled water was stirred for 12 h at ambient temperature. The suspension was allowed to stand for phase separation and was subsequently centrifuged to isolate the precipitate. The solid product was thoroughly characterized using a Thermo Fisher Scientific K-Alpha XPS instrument to investigate its surface composition and chemical states.

#### 2.3.4 Adsorption rate calculation

For the enzymatic activity analysis, 10 mg of each modified enzyme (laccase and catalase) was separately dissolved in 1 mL of distilled water. The treatment group was supplemented with 10 mg of cerium oxide, whereas the control group was maintained without cerium oxide. Both groups were stirred continuously for 1 h at room temperature. They were then centrifuged (10,000 × *g*, 10 min) to separate the precipitates, which were collected for enzymatic activity assessment.

The laccase activity was quantified using the guaiacol oxidation method (Umar, 2021). One unit of enzyme activity (U) was defined as the amount of enzyme required to oxidize 1 μmol of guaiacol per min under standard assay conditions. Catalase activity was determined using sodium thiosulfate titration (Zhu and Han, 2010). Similarly, one unit of activity was corresponded to the oxidation of 1 μmol of substrate per minute.

#### 2.3.5 Stability of laccase and catalase in cerium oxide

To investigate the stability of laccase and catalase in cerium oxide, 50 mL of each enzyme solution was supplemented with either 1% or 20% (w/v) cerium oxide. The control group contained 50 mL of the enzyme solution. The enzymatic activity in all groups was monitored at 48 h intervals throughout the experiment. The temporal evolution of enzymatic activity was analyzed by plotting the data with time (h) as the independent variable (abscissa) and relative enzyme activity (%) as the dependent variable (ordinate).

### 2.4 Additive manufacturing of the microchannel network catalyst bed

The three-dimensional microchannel network catalyst bed was fabricated using cerium oxide and titanium dioxide. This catalytic structure can optimize enzyme loading and substrate accessibility.

## 3 Results and discussion

### 3.1 The generation of hydrogen peroxide

The hydrogen peroxide content of each group was quantified relative to the 1% hydroquinone benchmark. EPR was performed to detect whether hydrogen peroxide radicals were present during the reaction.

As shown in Table 2, while the treatment group exhibited a higher hydrogen peroxide concentration compared to the control group, the absolute concentration in the treatment group remained relatively low, presenting a challenge for subsequent optimization. To elucidate the underlying reaction mechanism, EPR was used to detect the generation of hydrogen peroxide radicals during the reaction.

**TABLE 2:** Percentage concentration of H_2_O_2_ from hydroquinone catalyzed by different types of enzymes.

Substrate	Types of enzymes	Reaction temperature	Reaction hour	Content of the enzyme in the reaction solution	Relative concentration of H_2_O_2_
1% hydroquinone	​	4 °C	24 h	​	0%
1% hydroquinone	Free laccase	4 °C	24 h	9 U·mL^-1^	20.75% ± 1.1%
1% hydroquinone	Modified laccase	4 °C	24 h	9 U·mL^-1^	24.56% ± 1.37%
1% hydroquinone	Immobilized laccase	4 °C	24 h	9 U·mL^-1^	191.79% ± 5.76%

The time-dependent evolution of the radical species during laccase-catalyzed hydroquinone oxidation was systematically investigated using freeze-trapping EPR spectroscopy. Initial characterization revealed a distinct radical signal emerging after 5 min of reaction, with a g-factor of 2.004 and a characteristic peak-to-valley width of 9 G, which was unambiguously assigned to the hydroquinone radical species. A prolonged reaction time of 15 min led to a notable spectral shift, yielding an asymmetric signal identified as the p-hydroxycatechol radical (Figure 1a), indicating progressive oxidation of the initial radical species.

**FIGURE 1:**
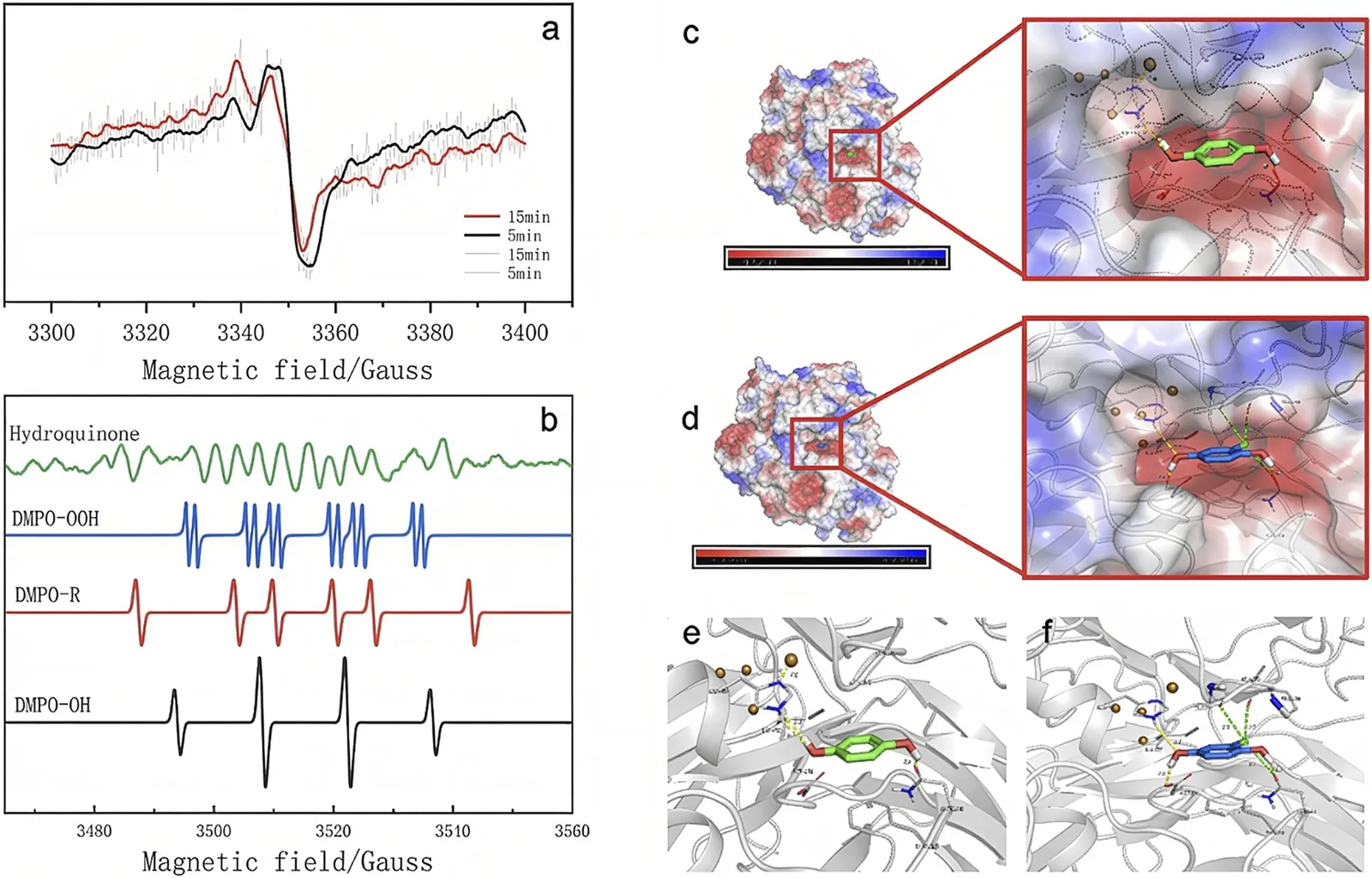
EPR spectra in **(a)** and **(b)**. X-band (9.39 GHz) EPR spectra of hydrogen peroxide radicals generated by laccase-catalyzed hydroquinone through freezing trapping in 5 min and 15 min **(a)**. Room temperature X-band (9.86 GHz) EPR spectra of radicals ( DMPO−OOH , DMPO−R , and DMPO−OH ) spin-trapped by DMPO in the presence of hydroquinone **(b)**. Schematic diagram of banding between active centers of laccase and hydroquinone or 2-chloro-hydroquinone in **(c)**, **(d)**, **(e)**, and **(f)**. Schematic diagram of laccase protein and 2-chloro-hydroquinone binding and enlarged view of the local area **(c)**. Schematic diagram of laccase protein and hydroquinone binding and enlarged view of the local area **(d)**. Schematic diagram of the interaction between hydroquinone **(e)**. Schematic diagram of the interaction between 2-chloro-hydroquinone **(f)**.

Spin trapping experiments successfully identified four distinct radical intermediates. The DMPO-OH adduct radical was characterized by isotropic hyperfine coupling constants (hfcs) of aN = aHβ = 14.8 G, producing the characteristic 1:2:2:1 quartet pattern. The DMPO-R adduct radical displayed a six-line hyperfine pattern with hfcs of aN = 16.5 G and aHβ = 22.9 G. The DMPO-OOH radical species was identified by its characteristic hfcs (aN = 14 G, aHβ = 10 G, and aHγ = 1.5 G). A comparative analysis of the EPR spectra of hydroquinone and its three radical analogs (Figure 1b) provided strong evidence of the presence of multiple radicals, including carbon-centered radicals and hydrogen peroxide radicals, through laccase-mediated catalysis. The suspected generation of hydroxyl radicals may be attributed to the superposition and cancellation of peak signals. This detailed radical characterization offers new insights into the complex oxidative transformation pathways of hydroquinone under enzymatic conditions.

Various hydroquinone derivatives, including 2-chloro-hydroquinone, are present in *P. occipitalis*. Structural analysis of the laccase-substrate complex revealed that both hydroquinone and 2-chloro-hydroquinone were strategically positioned within the hydrophobic pocket of the enzyme (Figures 1c,d), consistent with previous results. A detailed examination of the binding interactions showed that hydroquinone forms three specific hydrogen bonds: two with HIS458 and one with ASN206 (Figure 1e). The binding mode of 2-chloro-hydroquinone presents a more complex interaction network. The hydroxyl oxygen establishes hydrogen bonds with HIS458 and ASP206, whereas the para-hydroxyl oxygen forms an additional hydrogen bond with ASN264. The chlorine substituent engages in multiple halogen bonding interactions with GLY392, PRO394, and ASN264 (Figure 1f), with precise interatomic distances, as detailed in Table 3. Binding free energy calculations revealed a stronger affinity for the chlorinated derivative, with values of −4.6 kcal·mol^-1^ for hydroquinone and −5.1 kcal·mol^-1^ for 2-chloro-hydroquinone. This enhanced binding, coupled with the observed interaction networks, provides a structural basis for the rapid generation of hydrogen peroxide in *P. occipitalis*.

**TABLE 3:** Interaction data between laccase protein and hydroquinone or 2-chloro-hydroquinone.

Category	Interaction	Receptor residue ID	Distance (Å, noH)
Hydroquinone	Hydrogen bond	HIS458	2.3
		HIS458	2.6
		ASN206	2.4
2-Chloro-hydroquinone	Hydrogen bond	HIS458	2.5
		ASP206	2.2
		ASN264	2.5
	Halogen bonds	GLY392	3.5
		PRO394	3.5
		ASN264	3.3

The catalytic cycle of hydroquinone oxidation by laccase proceeds via a well-defined radical-mediated mechanism. The initial enzymatic oxidation of hydroquinone in the presence of H_2_O generates p-hydroxycatechol as the primary intermediate. This product subsequently coordinates with the T1 copper center of laccase, facilitating the formation of hydrogen peroxide radicals. The reaction pathway diverges based on the site-specific generation of hydrogen peroxide radicals. (i) When radical formation occurs at the C4 position, subsequent laccase-catalyzed oxidation yields m-hydroxy-p-benzoquinone as the final product. (ii) Alternatively, radical generation at the C3 position initiates a catalytic cycle involving H_2_O, leading to the continuous production of hydrogen peroxide radicals (Scheme 1).

**SCHEME 1:**
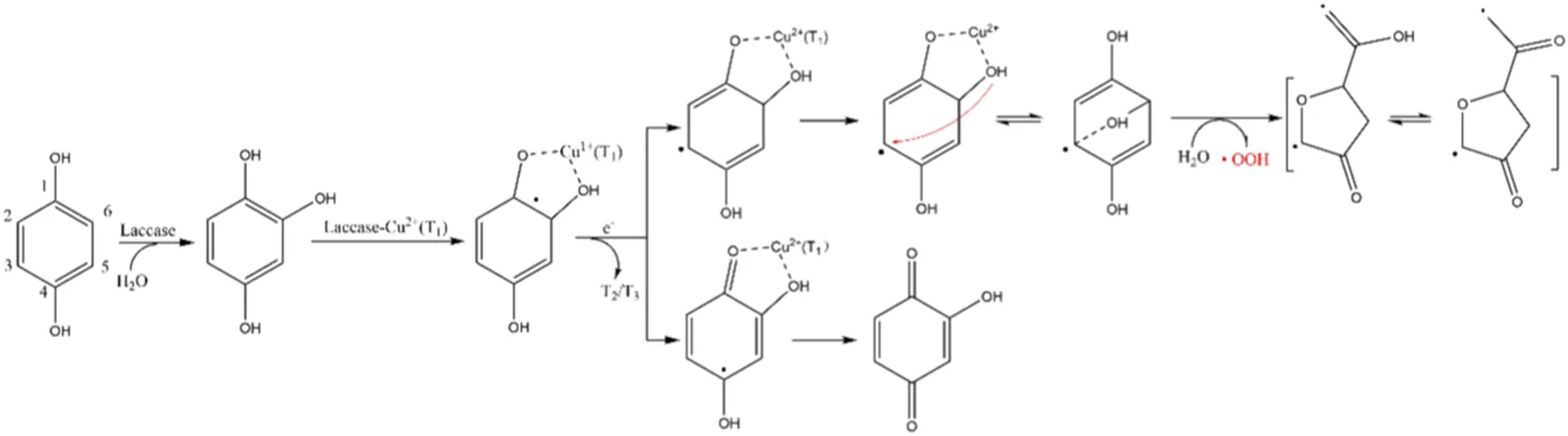
Mechanism of hydrogen peroxide radicals generated by laccase-catalyzed hydroquinone.

As established in previous studies, the complete reduction of 1 mol O_2_ to 1 mol H_2_O requires four electrons (Quintanar et al., 2010). However, only one electron is consumed per hydroperoxyl radical (·OOH) formed in the aforementioned side reaction pathway. Thus, 1 mol of O_2_ can accept 1 mol of electrons to generate 1 mol of OOH radicals; then, 2 mol of OOH radicals can produce 1 mol of H_2_O_2_ and 1 mol of O_2_. Furthermore, substrate radicals generated by laccase catalysis can trigger the homolytic cleavage of water molecules to produce hydroxyl radicals (·OH) and hydrogen atoms (H·) (Chen et al., 2017; Wang et al., 2020). The mutual collision of hydroxyl radicals subsequently generates hydrogen peroxide, constituting a secondary oxygen-independent hydrogen pathway for peroxide generation within the system. Additionally, 1 mol of H_2_O_2_ is then further decomposed into 0.5 mol of O_2_ and 1 mol of H_2_O molecule by co-immobilized catalase. From the perspective of local microenvironment turnover, for every 1 mol of O_2_ consumed by laccase in the single-electron pathway, a total of 1.5 mol of O_2_ are regenerated (1 mol from OOH disproportionation and 0.5 mol from H_2_O_2_ decomposition). This rapid oxygen regeneration creates a local oxygen-enriched microenvironment, a particularly significant effect given that under standard temperature and pressure (0 °C and 101.325 kPa), the maximum solubility of O_2_ in pure water is only approximately 14.62 mg·L^-1^ (48.9 mL·L^-1^ or 0.457 mmol·L^-1^), and this value decreases further with increasing temperature (e.g., 8.24 mg·L^-1^ at 25 °C).

Importantly, this cycle obeys thermodynamic laws. Initially, as the reaction proceeds and the oxygen in the liquid phase is continuously consumed, the mass transfer of oxygen from the gas phase to the liquid phase is thermodynamically spontaneous. Then, its energy derived from semi-quinone oxidation to benzoquinone propels the system continuously to consume hydroquinone and O_2_, ultimately producing benzoquinone, phenol, H_2_O_2_, and possibly hydrogen.

Beyond the oxygen regeneration cycle, a critical hydrogen element turnover mechanism operates synergistically to enhance the overall atomic economy of the system. In this reaction, hydrogen atoms are derived from two independent sources, hydroquinone molecules and solvent water molecules, confirming that water functions not only as a reaction medium but also as an essential substrate participating directly in the catalytic cycle. This conclusion is strongly supported by our previous study on laccase-catalyzed catechol ring-opening (Chen et al., 2017), which demonstrated that laccase can catalyze the homolytic cleavage of water molecules to generate hydroxyl radicals during oxidation. These hydroxyl radicals further dissociate to produce hydrogen atoms, which initiate radical chain reactions. Specifically, the generated hydrogen atoms recombine with hydroquinone semiquinone radicals to regenerate hydroquinone molecules, thereby forming a closed hydrogen turnover cycle. As the reaction proceeds, both hydroquinone and water are continuously consumed; however, the hydrogen-atom-mediated chain reaction effectively extends the lifetime of the reactive intermediates and reduces the net consumption of hydroquinone. This unique hydrogen cycling feature significantly improves the energy conversion efficiency of the system and reduces the overall substrate costs.

This bifurcated mechanism, supported by both experimental evidence and theoretical calculations, provides a comprehensive picture of complex radical-mediated transformations in laccase-catalyzed hydroquinone oxidation. Collectively, these findings offer the first experimental confirmation that H_2_O_2_ is generated via laccase-catalyzed hydroquinone oxidation through radical intermediates, resolving the long-standing debate on the source of H_2_O_2_ in the *P. occipitalis* chemical defense system. The proposed oxygen regeneration and hydrogen element turnover cycles also enrich the theoretical understanding of laccase-mediated radical catalysis. Given the insufficient stability and low catalytic efficiency of native free enzymes, further modifications and immobilization strategies are required to adapt this enzymatic reaction to practical energy-supply scenarios.

### 3.2 The immobilization of laccase and catalase

To address the stability challenges of free enzymes, a comprehensive stabilization strategy combining chemical modification and physical immobilization was implemented. In this study, laccase and catalase were modified with mPEG and immobilized on the catalyst bed. The immobilization results were analyzed using TEM and XPS, and the adsorption and stability of cerium dioxide were detected.

TEM was utilized to conduct a comprehensive three-dimensional morphological analysis focusing on the surface roughness and nano-structural features. Figure 2a presents the original structure of the nanoscale cerium oxide, which served as a reference for subsequent comparative analysis. A comparative examination of Figures 2b,c reveals distinct morphological changes characterized by the appearance of globular protrusions on the cerium oxide surface. These structural modifications provide direct visual evidence of the successful and effective immobilization of laccase and catalase enzymes on the cerium oxide supports. Globular features are absent in the original cerium oxide, confirming the establishment of stable enzyme-support interactions at the nanoscale level.

**FIGURE 2:**
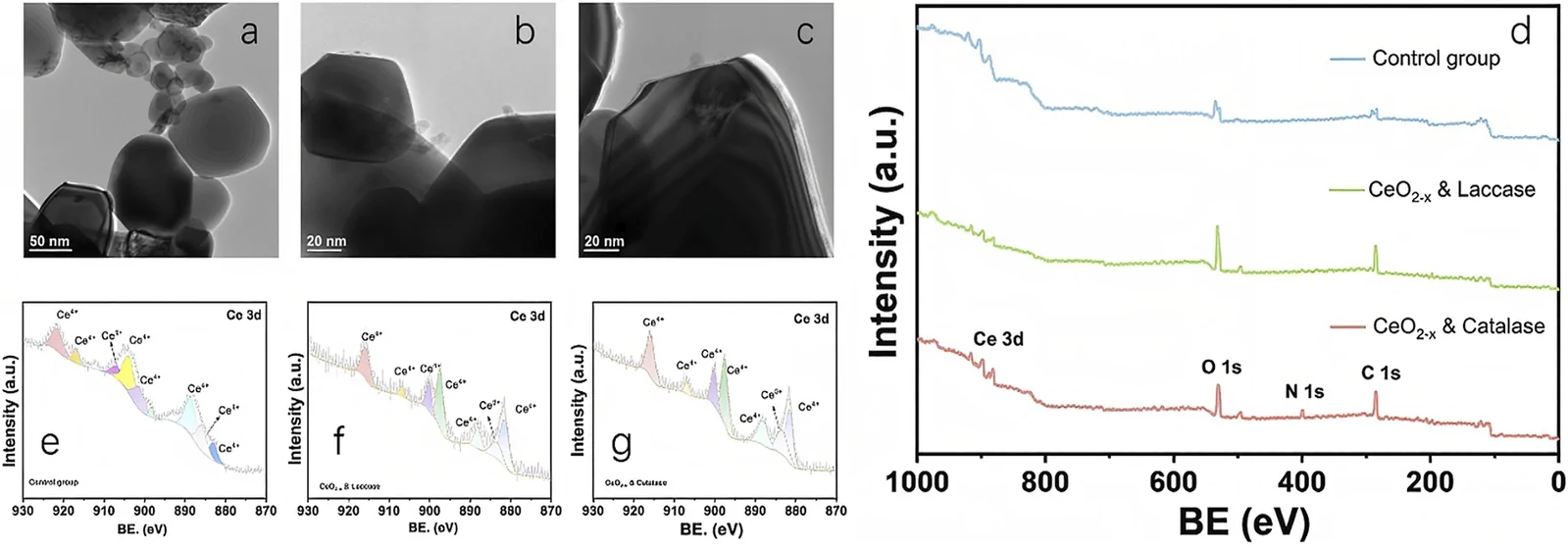
TEM in **(a–c)**. TEM of CeO_2-x_ **(a)**. TEM of CeO_2-x_ and laccase **(b)**. TEM of CeO_2-x_ and catalase **(c)**. XPS in **(d–g)**. Full spectrum scan of the control group, CeO_2-x_ and laccase and CeO_2-x_ and catalase **(d)**. Extended scans of Ce 3d for the control group, CeO_2-x_ and laccase, and CeO_2-x_ and catalase in **(e–g)**, respectively.

XPS analysis provided comprehensive evidence for the successful immobilization of enzymes on the CeO_2-x_ nanostructures. The full spectrum scan (Figure 2c) revealed the presence of characteristic elements, with Ce and O as the principal elements in the control group, and the emergence of C and N as additional characteristic elements in both the CeO_2-x_ and laccase and CeO_2-x_ and catalase samples. These findings are consistent with the TEM observations, confirming successful enzyme immobilization. High-resolution Ce 3d spectra (Figures 2d–f) demonstrated a significant increase in the Ce^4+^/Ce^3+^ ratio upon enzyme immobilization, providing strong evidence of successful integration of enzymes with the CeO_2-x_ support. Detailed analysis of the C1s spectrum in CeO_2-x_ and laccase revealed characteristic peaks at 291.48 eV (O–C=O), 284.83 eV (C–C), and 283.28 eV (C=O) (Supplementary Figure S1a), while CeO_2-x_ and catalase showed peaks at 288.18 eV (O–C=O), 286.28 eV (C=O), and 284.83 eV (C–C) (Supplementary Figure S1b). The presence of N1s in both the enzyme-immobilized samples (Supplementary Figures S1c,d) originated from the amino groups of the enzymes, with peak intensities reflecting the strength of the CeO_2-x_–enzyme interactions. Further confirmation was obtained from the O1s spectra, where the peak areas in CeO_2-x_ and laccase and CeO_2-x_ and catalase significantly exceeded those in the control group (Supplementary Figures S1e–g). These comprehensive XPS analyses, combined with TEM observations, provide robust evidence for the effective immobilization of laccase and catalase on the CeO_2-x_ nanostructures.

The adsorption efficiencies of laccase and catalase on cerium oxide were determined to be 38.89% and 72.78%, respectively (Supplementary Table S1). Both enzymes maintained their fundamental catalytic activities upon immobilization, demonstrating the structural stability of the enzyme–cerium oxide complexes (Supplementary Figure S2). However, temporal fluctuations in enzymatic activity were observed across all experimental groups, which may be attributed to several factors, including temporal effects, temperature variations, and inherent experimental uncertainties. The half-life of the enzymatic activity of the immobilized laccase and catalase reached 6 h–8 h, demonstrating significantly enhanced operational stability compared with their free counterparts.

Building on the foundational work on cerium redox states in superoxide dismutase mimetic activity (Yang et al., 2024), a composite catalyst bed was developed by integrating cerium redox chemistry, enzymatic catalysis, and titanium dioxide photosensitization. This synergistic combination leverages the unique properties of each component: cerium oxide for its redox activity, enzymes for their catalytic specificity, and titanium dioxide for its capacity to generate radicals. However, the laccase-catalyzed process generates free radicals, and thermal release during catalase-mediated catalysis can damage the native structure of the enzyme. Conversely, cerium oxide and titanium oxide exert dual protective and promotional effects; they not only enhance the electron/ion transfer efficiency at enzyme active centers via oxygen vacancies but also maintain the spatial structural rigidity of immobilized enzymes, thereby significantly reducing the rate of protein denaturation. The catalytic promotion of cerium oxide stems from its unique reversible Ce^4+^/Ce^3+^ redox cycle, which also exerts multiple auxiliary catalytic functions: In laccase-catalyzed hydroquinone oxidation to p-benzoquinone, released electrons are accepted by Ce^4+^, which is reduced to Ce^3+^ to drive the forward reaction. During product recycling, Ce^4+^ simultaneously facilitates the reduction of p-benzoquinone. In hydrogen peroxide decomposition, oxygen-deficient CeO_2-x_ absorbs released electrons and is oxidized back to Ce^4+^, thereby accelerating H_2_O_2_ breakdown. The oxygen vacancies within cerium oxide facilitate gas exchange (Esch et al., 2005). The catalyst bed design prioritizes high catalytic decomposition efficiency, drawing inspiration from the fractal dendriform geometry observed in the microchannel network of *P. occipitalis*. This biomimetic approach incorporates a hierarchical microchannel network with varying diameters and branching patterns established through additive manufacturing techniques (Figures 3a,b). Bioinspired designs offer several advantages: enhanced fluid distribution uniformity through optimized channel geometry, reduced flow resistance by imitating natural fluid transport systems, and improved mass transfer efficiency via fractal-based network optimization. This innovative design builds upon established principles of microchannel network optimization (Luo and Tondeur, 2011; Privat et al., 2017; Tondeur et al., 2009; Wu, 2012), while incorporating biological insights from insect microfluidics. The resulting catalyst bed structure represents a significant advancement in catalytic system design, combining biomimetic principles with advanced material engineering to optimize the decomposition performance.

**FIGURE 3:**
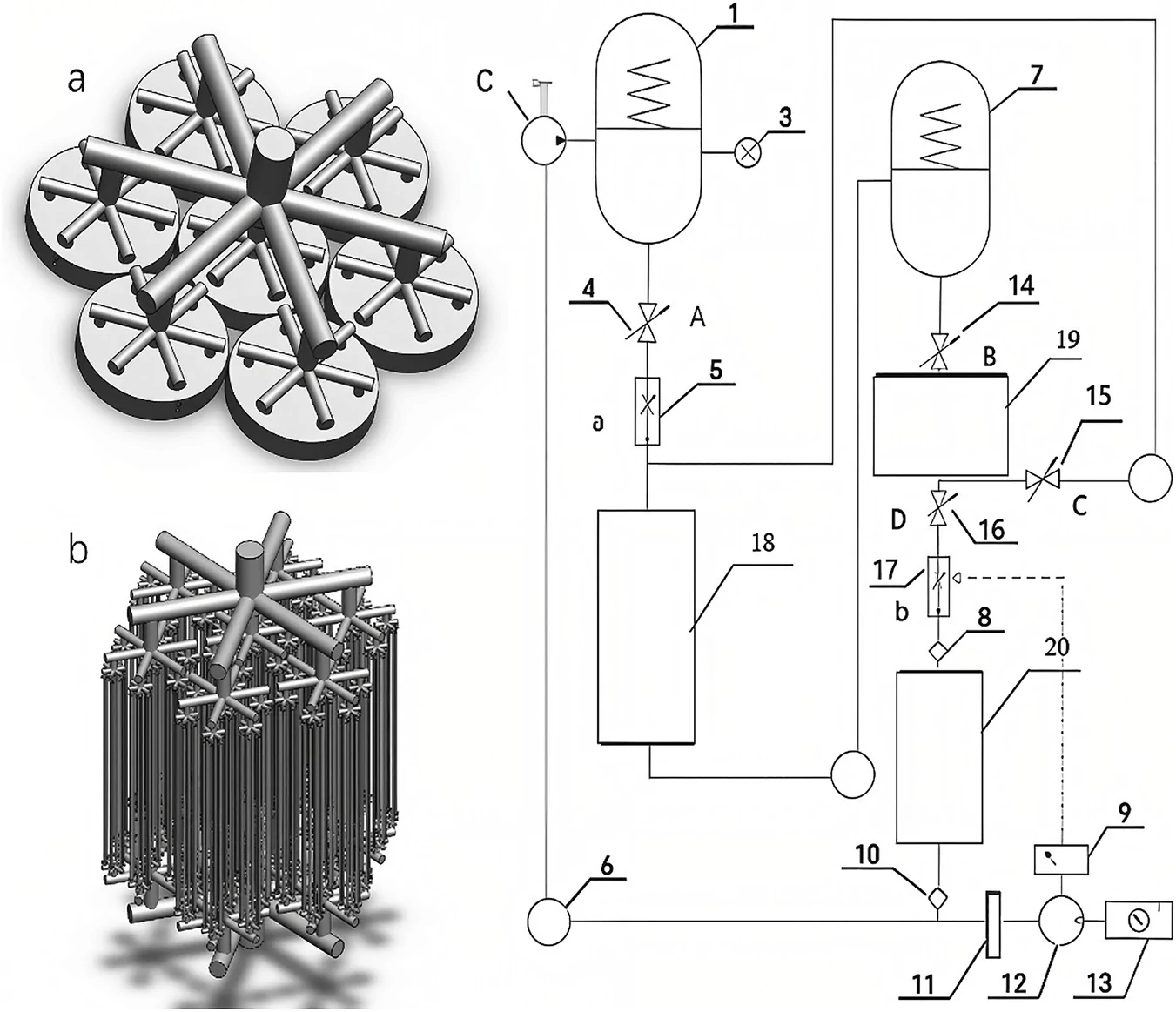
Construction of the catalyst bed in **(a–b)**. Pulse cascade supercharging system in **(c)**. 1, Hydroquinone reservoir; 2, fuel injection pump; 3, pressure gauge; 4, first stop valve; 5, first proportional throttle valve; 6, pump; 7, hydrogen peroxide reservoir; 8, low-pressure check valve; 9, speed sensor; 10, high-pressure check valve; 11, PTFE membrane; 12, pneumatic motor; 13, load; 14, second stop valve; 15, third stop valve; 16, fourth stop valve; 17, second proportional throttle valve; 18, laccase catalyst bed; 19, hydrogen peroxide concentration sensor; 20, catalase catalyst bed.

Overall, mPEG-modified laccase and catalase were successfully co-immobilized on cerium oxide–titanium dioxide composite supports, as confirmed by TEM and XPS. The adsorption efficiency and operational stability of the enzymes were quantitatively evaluated, and the immobilized enzymes reached a half-life of 6 h–8 h, significantly outperforming their free counterparts. Unlike conventional enzyme immobilization strategies that only serve as physical supports, the CeO_2_/TiO_2_ composite in this study exerts a dual function of structural protection and auxiliary catalysis: the reversible Ce^4+^/Ce^3+^ redox cycle acts as an electron shuttle to accelerate the catalytic reaction, and abundant oxygen vacancies promote gas mass transfer. This synergistic immobilization strategy achieves a 9.2-fold increase in H_2_O_2_ yield relative to free laccase, providing an efficient and stable biocatalytic platform for subsequent biomimetic energy system construction.

### 3.3 Design of the catalyst bed and pulse cascade supercharging system

The evolutionary development of organisms manifests not only in structural morphology and environmental adaptation but also in sophisticated energy acquisition, transformation, and utilization mechanisms. These biological systems achieve remarkable functionality through optimization based on the principle of minimum energy expenditure. However, conventional dynamic systems have inherent limitations in terms of the fuel concentration and system mass efficiency. Inspired by the morphological and energetic flow characteristics of *P. occipitalis*, a novel approach that significantly reduces upstream pressure requirements and enhances energy conversion efficiency was developed (Yang, 2016). Building on the pioneering work on the selective directional liquid transport mechanisms observed in *Crassula muscosa* by Yang et al. (2024), a unidirectional liquid transport system was implemented in our pulse cascade supercharging system.

The operational sequence of the pulse cascade supercharging system is shown in Figure 3c. The system boots when the hydrogen peroxide volume falls below a predetermined threshold, triggering the opening of the first stop valve. The first proportional throttle valve precisely regulates the flow rate and closes upon reaching a specified hydroquinone output volume. Subsequently, the hydroquinone solution enters the internal circulation loop through sequential activation of the second and third stop valves. This circulation pathway comprises a laccase catalyst bed, a hydrogen peroxide reservoir, and a hydrogen peroxide concentration sensor, and then returns to the catalyst bed, forming a closed loop. The internal circulation terminates when the hydrogen peroxide concentration sensor detects the preset concentration value, prompting the closure of the second and third stop valves. During the gas generation phase, the second and fourth stop valves activate, directing the hydrogen peroxide solution into the catalase catalyst bed at a controlled rate regulated by the second proportional throttle valve. A dedicated condensing apparatus maintains thermal equilibrium by absorbing reaction enthalpy, thereby stabilizing the system temperature within the optimal range of 25 °C–30 °C. When the gas pressure exceeds the designated threshold, the high-pressure check valve engages, thereby enabling product discharge. The gaseous output passes through a polytetrafluoroethylene membrane filter, which effectively prevents liquid passage before reaching the air motor. The motor precisely regulates the gas flow into the storage chamber via proportional throttle valve B. The liquid byproducts, consisting of water and benzoquinone, are efficiently recycled to the hydroquinone reservoir, where benzoquinone undergoes facile reduction to regenerate hydroquinone for subsequent reaction cycles.

Compared with traditional H_2_O_2_ power systems that require pre-storage of high-concentration H_2_O_2_, the pulsed cascade supercharging system proposed in this work realizes on-demand generation and *in situ* utilization of H_2_O_2_ through compartmentalized cascade catalysis, fundamentally eliminating the safety risks of high-pressure storage and transportation of concentrated H_2_O_2_. The fractal microchannel catalyst bed, inspired by the biological microchannel network of *P. occipitalis*, also effectively improves fluid distribution uniformity and mass transfer efficiency, offering a new design concept for high-efficiency biomimetic catalytic reactors.

## 4 Conclusion

Biomimetic biocatalysis inspired by natural biological systems is a promising paradigm for advancing clean energy conversion technologies. Among natural defensive systems, *P. occipitalis* has long garnered research interest for its exceptional ability to generate and utilize H_2_O_2_ under physiological conditions, yet the biosynthetic origin of H_2_O_2_ in its glands has remained a long-standing mechanistic puzzle. In this study, we systematically elucidate that laccase catalyzes the oxidation of hydroquinone to generate H_2_O_2_ via radical intermediates and further reveal a triple synergistic turnover cycle that underpins the high efficiency of the reaction. Building on this mechanistic foundation, we construct a compartmentalized biomimetic energy-supply system that addresses the key limitations of conventional power devices.

Distinct from the canonical laccase catalytic paradigm, in which O_2_ undergoes four-electron reduction to water at a single copper active site with substrate transformation occurring in the same binding pocket, the reaction system described here operates through a spatially coupled radical-mediated mechanism. The self-sustaining oxygen regeneration cycle creates a local oxygen-enriched microenvironment, fundamentally decoupling the enzymatic activity from the dissolved oxygen concentration in aqueous solutions. The hydrogen element turnover cycle, which uses both hydroquinone and solvent water as hydrogen sources, significantly improves the atomic economy and reduces net organic substrate consumption. Additionally, the reversible Ce^4+^/Ce^3+^ redox couple of cerium oxide functions as an efficient electron shuttle that accelerates both laccase-catalyzed oxidation and H_2_O_2_ decomposition, while abundant surface oxygen vacancies promote gas mass transfer. This triple-cycle cooperative mode represents a novel catalytic strategy for laccase-mediated H_2_O_2_ production.

From a technical perspective, conventional enzyme immobilization strategies primarily serve as physical supports to improve stability, whereas industrial H_2_O_2_ production via the anthraquinone process is energy-intensive and leads to heavy pollutant discharge. In this study, we developed a co-immobilization strategy for laccase and catalase based on CeO_2_/TiO_2_ composite supports. The composite carrier exerts a dual function of structural protection and auxiliary catalysis; it maintains the conformational rigidity of immobilized enzymes to extend the operational half-life and simultaneously promotes interfacial electron transfer to boost catalytic performance. The immobilized laccase achieved a relative H_2_O_2_ yield of 191.79%, a 9.2-fold improvement over free laccase. This biocatalytic system operates under mild conditions with high substrate specificity, thereby avoiding toxic by-products and the particle leaching problem common to conventional heterogeneous catalysts.

Inspired by the compartmentalized anatomical structure of *P. occipitalis*, in which the precursor storage, catalytic reaction, and jet output are spatially separated, a fractal microchannel catalyst bed and pulsed cascade booster system were constructed. Unlike traditional H_2_O_2_ power devices that rely on pre-stored high-concentration H_2_O_2_ under high pressure, the integrated system realizes the cascade operation of on-demand H_2_O_2_ synthesis, *in situ* catalytic decomposition, and energy output. The spatial separation of H_2_O_2_ generation and utilization fundamentally eliminates the safety hazards associated with concentrated peroxide storage and transportation, while the biomimetic fractal microchannel design effectively improves fluid distribution uniformity and mass transfer efficiency. This design effectively addresses the inherent drawbacks of conventional energy systems such as high noise, low energy efficiency, and pollutant emissions.

Overall, this study not only deepens the fundamental understanding of insect chemical defense mechanisms and expands the catalytic repertoire of laccase-mediated radical reactions but also establishes a novel biomimetic catalytic paradigm for clean energy conversion. The proposed compartmentalized cascade strategy has broad application prospects for portable power supplies, soft robotics, and distributed micropower systems.

## Data Availability

The original contributions presented in the study are included in the article/Supplementary Material, further inquiries can be directed to the corresponding author.
