# Supplementary material for: Establishment of new energy supply construction inspired by the defensive mechanism of Pheropsophus occipitalis
Source: Front Chem. 2026 Oct 20;14:1831087. doi: 10.3389/fchem.2026.1831087 (PMC13729060; doi:10.3389/fchem.2026.1831087)
Supplement: Supplementary file 1 [file DataSheet1.pdf]

Supplyment materials

XPS

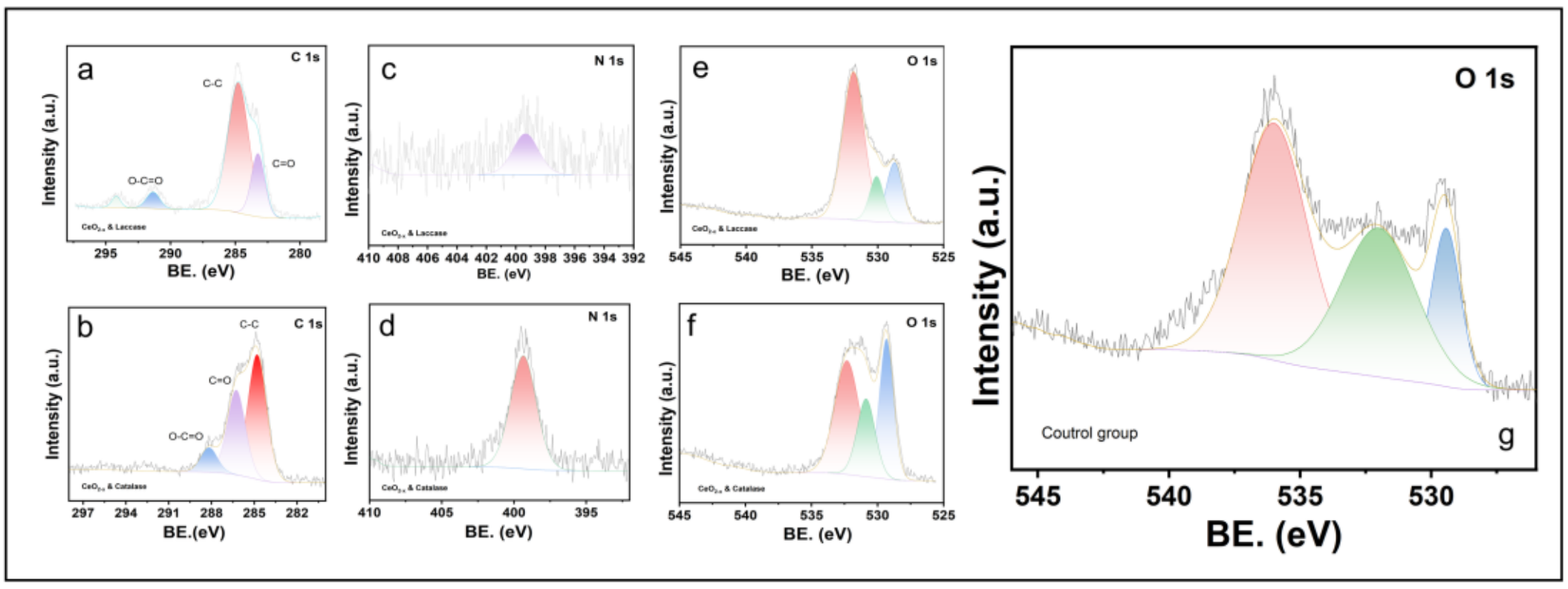

Fig. S1. The XPS of C in (a) and (b). The XPS of N in (c) and (d). The XPS of O in (e), (f) and (g).

Table S1

The adsorption rate of enzymes (laccase and catalase) in cerium oxide.

	Enzyme activity (with cerium oxide)	Enzyme activity (without cerium oxide)	Adsorption
Laccase	1555.68	4000.32	38.89%
Catalase	104800	144000	72.78%

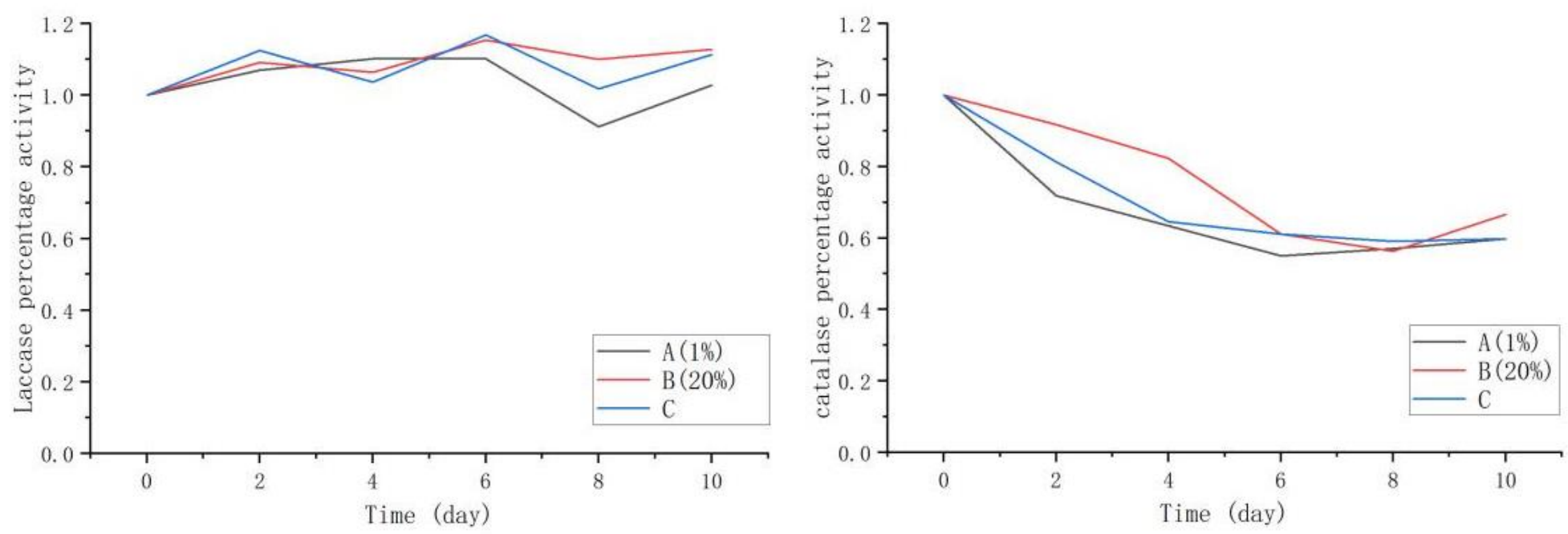

Fig. S2. The adsorption stability of enzymes (laccase and catalase) in cerium oxide.
